# Crystal structure of a hydrate of 1,3,5-substituted 2,4,6-tri­alkyl­benzene bearing hy­droxy­methyl and pyridinyl­amino groups

**DOI:** 10.1107/S2056989026008856

**Published:** 2026-09-08

**Authors:** Manuel Stapf, Wilhelm Seichter, Monika Mazik

**Affiliations:** a Institut für Organische Chemie, Technische Universität Bergakademie Freiberg, Leipziger Str. 29, 09599 Freiberg/Sachsen, Germany; Tokyo University of Science, Japan

**Keywords:** crystal structure, hydrogen bonding, hy­droxy­methyl group, hydrate, water cluster, supra­molecular synthons

## Abstract

The asymmetric unit of the title compound, (3,5-bis­{[(4,6-di­methyl­pyridin-2-yl)amino]­meth­yl}-2,4,6-tri­ethyl­phen­yl)methanol sesquihydrate, C_29_H_40_N_4_O·1.5H_2_O, **1**·1.5H_2_O, contains two crystallographically independent mol­ecules (*I* and *II*) and three water mol­ecules [(**1**)_2_(H_2_O)_3_]. These components are connected by O—H⋯O and N—H⋯O hydrogen bonds to form a 2:3 complex. Within this structural unit, the three water mol­ecules and one of the hy­droxy­methyl O atoms create a strongly distorted eight-membered *R*_4_^3^(8) supra­molecular synthon. In addition, the three water mol­ecules form a structure unit that can be defined as a *D*3 water cluster. In the crystal, neighbouring 2:3 complexes are linked by N—H⋯O and O—H⋯N hydrogen bonds into infinite strand-like aggregates extending along the *a-*axis direction. The pattern of non-covalent inter­molecular bonding is completed by numerous C—H⋯π arene inter­actions. The crystal contains highly disordered solvent mol­ecules that could not be refined to an acceptable level. Their contribution to the diffraction intensities was therefore removed using the BYPASS routine in *OLEX2*, and these solvent mol­ecules were excluded from the reported mol­ecular weight and crystal density.

## Abstract 2

In the asymmetric unit, two mol­ecules of the 1,3,5-substituted 2,4,6-tri­alkyl­benzene and three water mol­ecules are connected by hydrogen bonds, forming well-defined complexes that constitute the smallest supra­molecular entities.

## 1. Chemical context

Carbohydrate-binding proteins employ a variety of functional groups to achieve the selective recognition of carbohydrate substrates. Among these, the hydroxyl side chains of serine and threonine residues frequently contribute to binding inter­actions, as revealed by crystal structures of protein–carbohydrate complexes (Sauter *et al.*, 1992 ▸; Naismith & Field, 1996 ▸; Sharon & Lis, 2007 ▸). The mol­ecular recognition strategies adopted by such proteins have provided valuable inspiration for the design of artificial carbohydrate receptors (Davis & Wareham, 1999 ▸; Mazik, 2012 ▸; Manick *et al.*, 2023 ▸). In this context, hy­droxy groups have also been incorporated as structural elements for carbohydrate recognition in both acyclic (Mazik & Buthe, 2008 ▸; Mazik & Kuschel, 2008 ▸; Carrero *et al.*, 2013 ▸; Stapf *et al.*, 2023 ▸; Stapf & Mazik, 2026 ▸) and macrocyclic (Amrhein *et al.*, 2016 ▸; Amrhein & Mazik, 2021 ▸) receptor architectures. Furthermore, representatives of the acyclic receptors bearing hy­droxy groups have demonstrated the ability to participate in the mol­ecular recognition of substrates beyond carbohydrates, such as hydro­nium and hydroxide ions (Stapf *et al.*, 2015 ▸). This highlights their broader applicability in artificial receptor design.

The hy­droxy­methyl-containing compound described here belongs to the class of 1,3,5-substituted 2,4,6-tri­alkyl­benzene derivatives and was synthesized as part of our investigations into the relationship between structure and binding affinity in carbohydrate recognition.
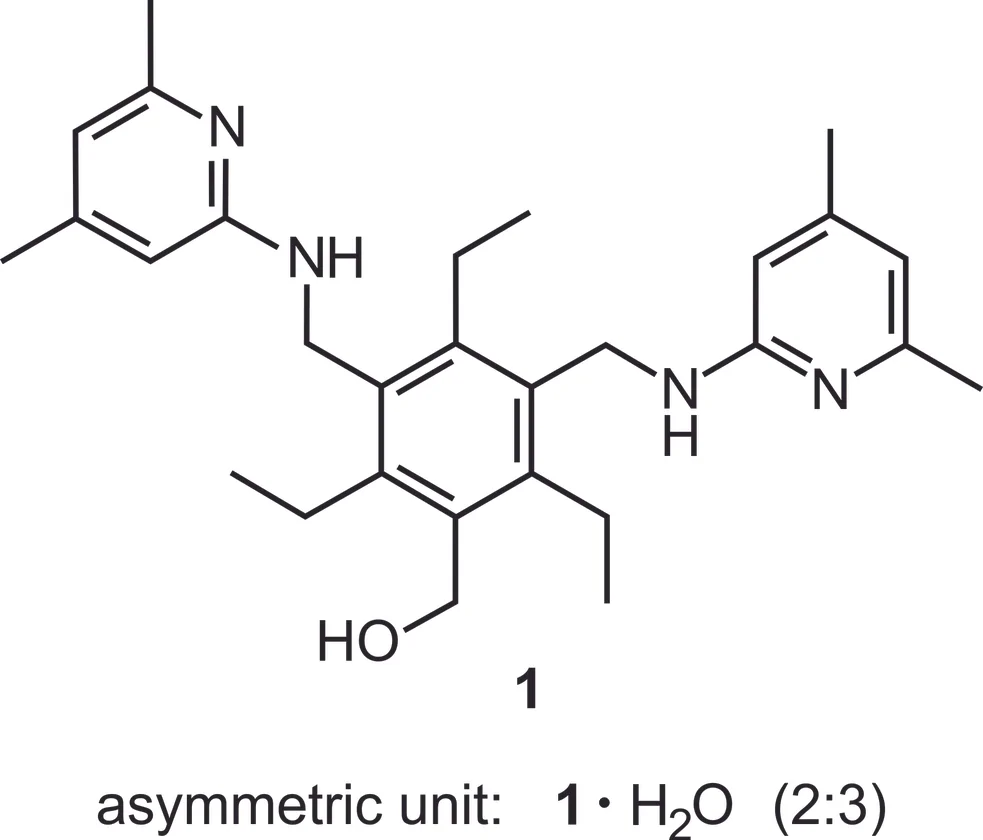


## 2. Structural Commentary

Compound **1** crystallizes from aceto­nitrile in the form of colourless platelets in the monoclinic space group *P*2_1_/*n*. The asymmetric unit comprises two crystallographically independent organic mol­ecules of **1** (labelled *I* and *II*) and three water mol­ecules, which are inter­connected through O—H⋯O and N—H⋯O hydrogen bonds in the manner shown in Fig. 1 ▸. Additionally, the crystal contains disordered solvent mol­ecules (aceto­nitrile) that could not be refined satisfactorily. Therefore, a modified data set was generated using the BYPASS routine (van der Sluis & Spek, 1990 ▸) of the *OLEX2* program, in which the contribution of the disordered mol­ecular species to the structure factors has been eliminated.

In the crystal, both mol­ecules *I* and *II* adopt a geometry in which the pyridinyl-containing side arms and the hy­droxy­methyl OH group are located on one side of the central aromatic ring, while the ethyl substituents are directed towards the opposite side, resulting in an alternating *ababab* arrangement (*a* = above, *b* = below; Das & Barbour, 2009 ▸; Koch *et al.*, 2017 ▸; Schulze *et al.*, 2017 ▸). Despite their overall structural similarity, the two independent mol­ecules differ slightly in their conformations. In the case of mol­ecule *I*, the inclination angles of the pyridine rings with respect to the plane of the benzene ring are 82.2 (1) and 87.6 (1)°, whereas the corresponding angles for mol­ecule *II* are 76.1 (1) and 82.8 (1)°. The dihedral angles between the pyridinyl units are 39.4 (1) and 48.2 (1)°, respectively. Although the functionalized side arms of the mol­ecules adopt an extended conformation, they exhibit different degrees of torsion. This is evident from the values for the torsion angles along the atomic sequences C_benz_—C—N—C_pyr_, which are 170.5 (1) and −162.3 (1)° for mol­ecule *I* and 176.3 (1) and 173.0 (1)° for mol­ecule *II*.

## 3. Supra­molecular features

The 2:3 complex of compound **1** and water mol­ecules, shown in Fig. 1 ▸, represents the basic supra­molecular unit of the crystal structure. Within this unit, the water mol­ecules form a type *D*3 cluster (Infantes & Motherwell, 2002 ▸; Infantes *et al.*, 2003 ▸). Hydrogen-bond formation between terminal hydrogen atoms of this cluster and the hy­droxy­methyl atom O2 of mol­ecule *II* results in formation of a cyclic synthon of O—H⋯O bonds [*d*(H⋯O) = 1.89 (2)–1.98 (2) Å; Table 1 ▸], the structure of which can be described by the graph set 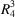
(8) (Etter, 1991 ▸; Bernstein *et al.*, 1995 ▸). Furthermore, the OH groups of the non-equivalent mol­ecules form an O—H⋯O bond with one another [*d*(H⋯O) = 1.78 (2) Å], while the amino hydrogen atoms of mol­ecule *I* are involved in the formation of N—H⋯O bonds [*d*(H⋯O) = 2.09 (1), 2.03 (2) Å] with the water oxygen atoms O4 and O5. Fig. 2 ▸ illustrates the linking of the complexes via hydrogen bonding resulting in the formation of an infinite strand-like mol­ecular aggregate that extends in direction of the crystallographic *a* axis. Its structure reveals that, in addition to the aforementioned eight-membered motif (light pink), other cyclic and chain-like synthons are present in the pattern formed by hydrogen bonds.

In the case of the ring synthon of the structure 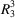
(8) (yellow), the amino hydrogen atom H7 and the ring nitro­gen atom N8 of a pyridinyl­amino group in mol­ecule *II* act as binding sites, so that this synthon contains an O—H⋯O [*d*(H⋯O) = 1.89 (2) Å], N—H⋯O [*d*(H⋯O) = 2.54 (2) Å] and an O—H⋯N hydrogen bond [*d*(H⋯N) = 1.93 (2) Å].

Another ring synthon [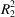
(6) (purple)] is formed by the second pyridinyl­amino group of this mol­ecule and comprises N—H⋯O [*d*(H⋯O) = 2.44 (2) Å] and O—H⋯N hydrogen bonds [*d*(H⋯N) = 1.94 (1) Å]. The chain-like paths of the hydrogen bonds marked in green and orange, can both be described by the graph set 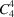
(8). In addition to the hydrogen bonds involving OH and NH groups, a large number of C—H⋯π inter­actions [*d*(H⋯*Cg*) = 2.71–2.98 Å; Nishio *et al.*, 1995 ▸], in which all arene units act as acceptors, contribute to the consolidation of the crystal structure.

The packing diagram shown in Fig. 3 ▸ indicates that the structure formed by the mol­ecules features channel-like voids extending along the crystallographic *a* axis, with an approximately elliptical cross-section profile. The walls of these voids are essentially defined by the less polar parts of the mol­ecules of **1**. These cavities accommodate the disordered solvent mol­ecules. The volume of the potentially solvent-accessible voids is 566 Å^3^ (9.3%) per unit cell, to which 122 electrons can be assigned. Fig. 4 ▸ shows the surface profile of the crystal voids.

Regarding the *D*3 water cluster mentioned above, it should be noted that such a discrete chain of three water mol­ecules has also been observed in the cavity of another 1,3,5-substituted 2,4,6-tri­alkyl­benzene containing pyridinyl and phenanthrolinyl groups (Mazik & Hartmann, 2008 ▸; Mazik *et al.*, 2009 ▸).

## 4. Database survey

The Cambridge Structural Database (CSD, Version 6.01, update February 2026; Groom *et al.*, 2016 ▸) was searched for fully alkyl-substituted benzene derivatives bearing at least one hy­droxy­methyl substituent. This search yielded eight hits, which were reduced to five relevant structures after excluding compounds containing metal ions.

Crystallization of 1,3,5-tris­(hy­droxy­meth­yl)-2,4,6-tri­ethyl­benzene from wet tetrahydrofuran (THF) affords an inclusion complex with a hydro­nium-hydroxide ion pair (CSD refcode BUGMAT; Stapf *et al.*, 2015 ▸). The hydroxyl groups of the host mol­ecule bind the ions through O—H⋯O hydrogen bonds, thereby generating a coordination environment comparable to the hydration sphere of water mol­ecules. Under modified crystallization conditions, however, the THF solvate of the same compound is obtained (BUGMEX; Stapf *et al.*, 2015 ▸). In contrast, the methyl-substituted analogue, 1,3,5-tris­(hy­droxy­meth­yl)-2,4,6-tri­methyl­benzene (BUGLUM; Stapf *et al.*, 2015 ▸), exhibits a lower degree of preorganization and crystallizes as a solvent-free structure. Hexa­kis­(hy­droxy­meth­yl)benzene (YICFIA; Rothenberger & Ponikiewski, 2007 ▸) adopts a crystal conformation in which three adjacent hy­droxy­methyl groups are oriented above and the remaining three below the plane of the benzene ring. This arrangement facilitates the formation of both intra- and, in particular, inter­molecular O—H⋯O hydrogen bonds. Likewise, the mol­ecules of 1,4-bis­(hy­droxy­meth­yl)-2,3,5,6-tetra­methyl­benzene (OJEZUY; Britton, 2003 ▸) are inter­connected by O—H⋯O hydrogen bonds to form a three-dimensional network.

## 5. Synthesis and crystallization

The synthesis of compound **1** was performed according to the literature (Mazik & Kuschel, 2008 ▸). Crystals suitable for single crystal X-ray diffraction were obtained by slow evaporation of aceto­nitrile from a solution of this compound at ambient temperature. The acetonitrile was used without prior drying, and the water molecules observed in the crystal structure can be attributed to residual water in the solvent. *Analysis data*: ^1^H NMR (500 MHz, CDCl_3_, ppm): *δ* = 1.22 (*t*, 3H, ^3^*J* = 7.5 Hz, CH_2_C**H**_3_), 1.24 (*t*, 6H, ^3^*J* = 7.5 Hz, CH_2_C**H**_3_), 2.23 (*s*, 6H, ArC**H**_3_), 2.35 (*s*, 6H, ArC**H**_3_), 2.74 (*q*, 2H, ^3^*J* = 7.5 Hz, C**H**_2_CH_3_), 2.84 (*q*, 4H, ^3^*J* = 7.5 Hz, C**H**_2_CH_3_), 4.20 (*br*, 2H, CH_2_N**H**), 4.37 (*d*, 4H, ^3^*J* = 4.2 Hz, C**H**_2_NH), 4.76 (*s*, 2H, C**H**_2_OH), 6.08 (*s*, 2H, Ar**H**), 6.34 (*s*, 2H, Ar**H**); ^13^C NMR (125 MHz, CDCl_3_, ppm): *δ* = 16.8 (CH_2_**C**H_3_), 17.0 (CH_2_**C**H_3_), 21.1 (Ar**C**H_3_), 22.8 (**C**H_2_CH_3_), 23.0 (**C**H_2_CH_3_), 24.2 (Ar**C**H_3_), 40.6 (**C**H_2_NH), 58.9 (**C**H_2_OH), 103.6 (Ar**C**), 113.9 (Ar**C**), 133.1 (Ar**C**), 134.8 (Ar**C**), 143.7 (Ar**C**), 144.1 (Ar**C**), 148.9 (Ar**C**), 156.6 (Ar**C**), 158.2 (Ar**C**); IR (ATR, cm^−1^): 3318, 2961, 2918, 2866, 1609, 1568, 1494, 1452, 1372, 1320, 1229, 1207, 1163, 1010, 981, 813, 751, 535.

## 6. Refinement

Crystal data, data collection and structure refinement details are summarized in Table 2 ▸. The non-hydrogen atoms were refined anisotropically. The positions of the hydrogen atoms bound to O and N were found in difference-Fourier maps and the O—H and N—H distances refined to a target value of 0.84 (1) and 0.89 (1) Å, respectively. All other hydrogen atoms were positioned geometrically and allowed to ride on their parent atoms: C—H = 0.95 Å for aryl H atoms, C—H = 0.99 Å for methyl­ene groups and C—H = 0.98 Å for methyl groups with *U*_iso_(H) = 1.5*U*_eq_(C) for methyl groups and *U*_iso_(H) = 1.2*U*_eq_(C) for other hydrogen atoms.

## Supplementary Material

Crystal structure: contains datablock(s) I. DOI: 10.1107/S2056989026008856/jp2032sup1.cif

Structure factors: contains datablock(s) I. DOI: 10.1107/S2056989026008856/jp2032Isup2.hkl

Supporting information file. DOI: 10.1107/S2056989026008856/jp2032Isup3.cml

CCDC reference: 2583267

Additional supporting information:  crystallographic information; 3D view; checkCIF report

## Figures and Tables

**Figure 1:**
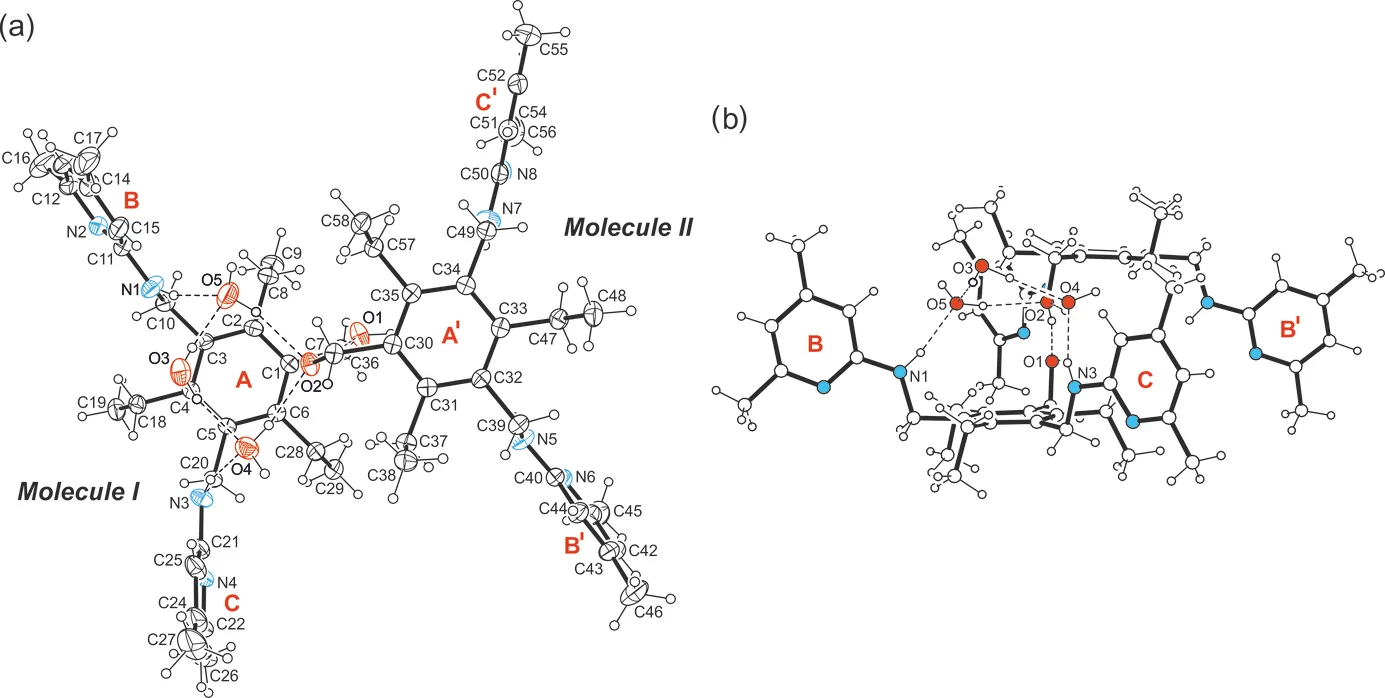
(*a*) Perspective view of the content of the asymmetric unit of the title compound including labelling of non-hydrogen atoms and ring specification (A–C, A′–C′). The displacement ellipsoids are drawn at the 50% probability level. (*b*) Ball-and-stick representation of the 2:3 complex of compound **1** and water mol­ecules. Broken lines represent hydrogen-bond inter­actions.

**Figure 2:**
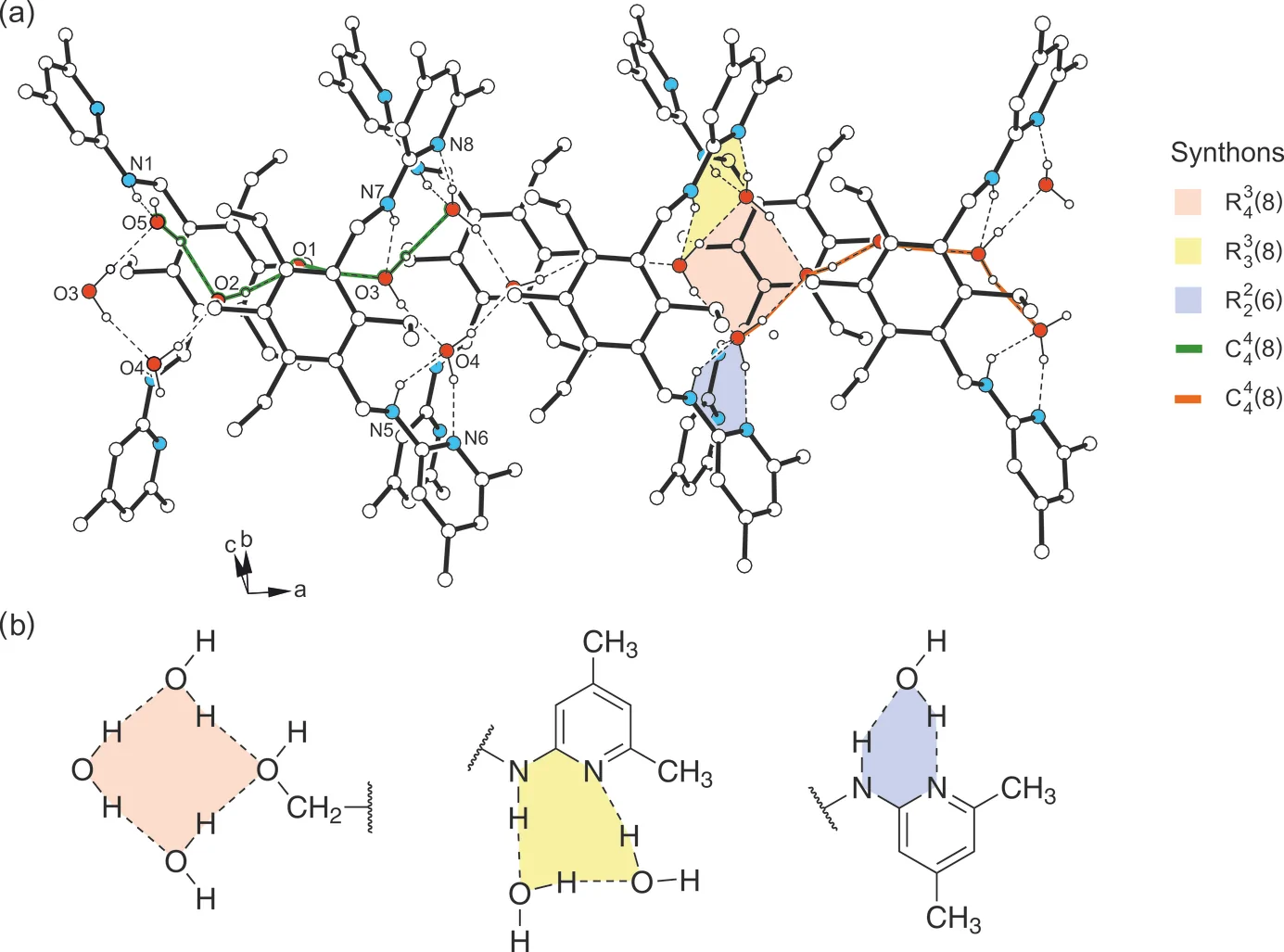
(*a*) Excerpt of the strand-like supra­molecular aggregates in the crystal structure of the title compound including the labelling of the acceptor atoms involved in hydrogen bonding. The supra­molecular synthons (rings, chains) are marked by colour highlighting. (*b*) Schematic illustration of the ring synthons. All of the hy­droxy­methyl and pyridinyl­amino groups shown here are part of mol­ecule *II*.

**Figure 3:**
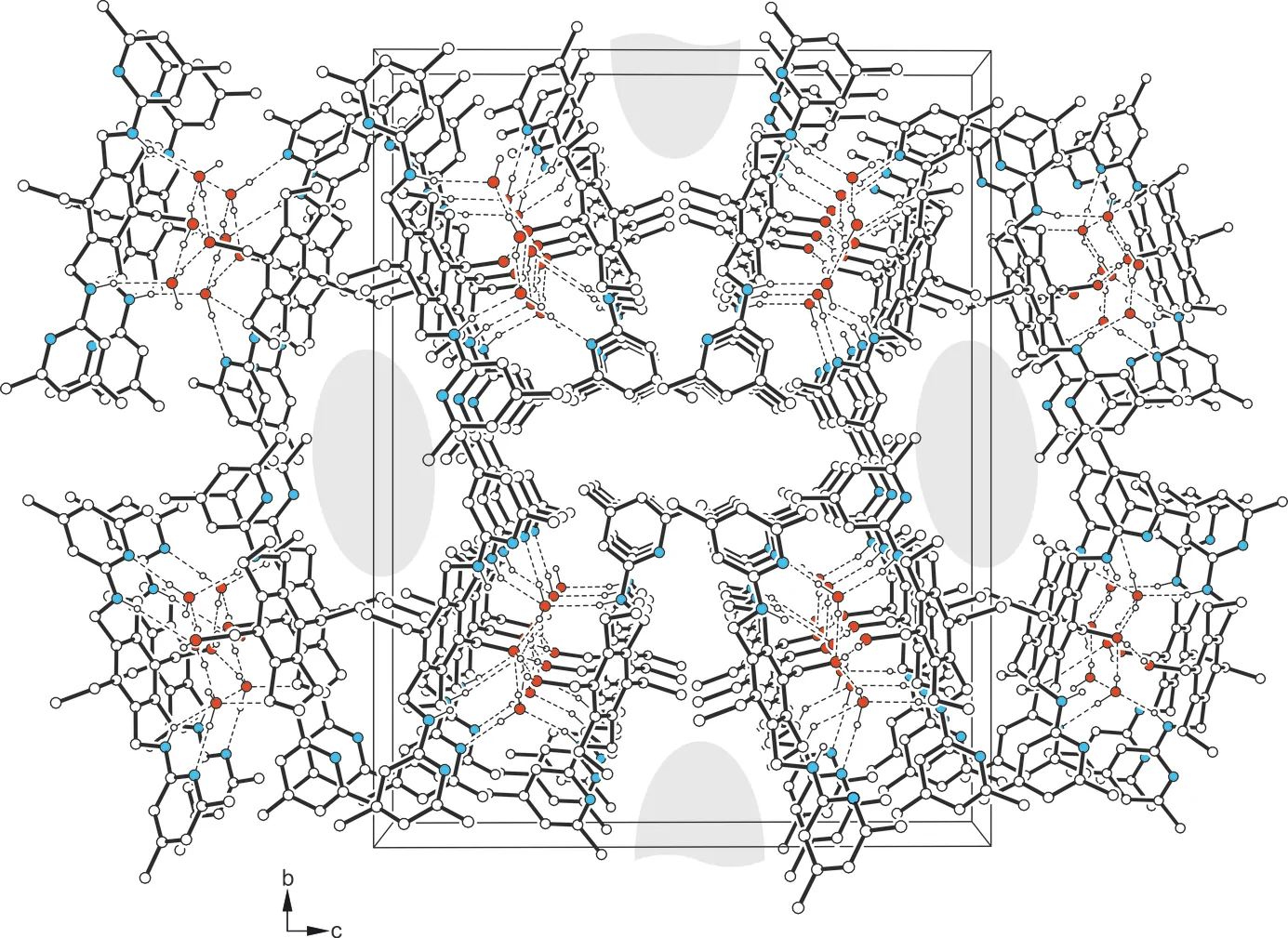
Packing diagram of the title compound viewed down the crystallographic *a* axis. Broken lines represent hydrogen-bond inter­actions. The channel-like voids are marked in grey.

**Figure 4:**
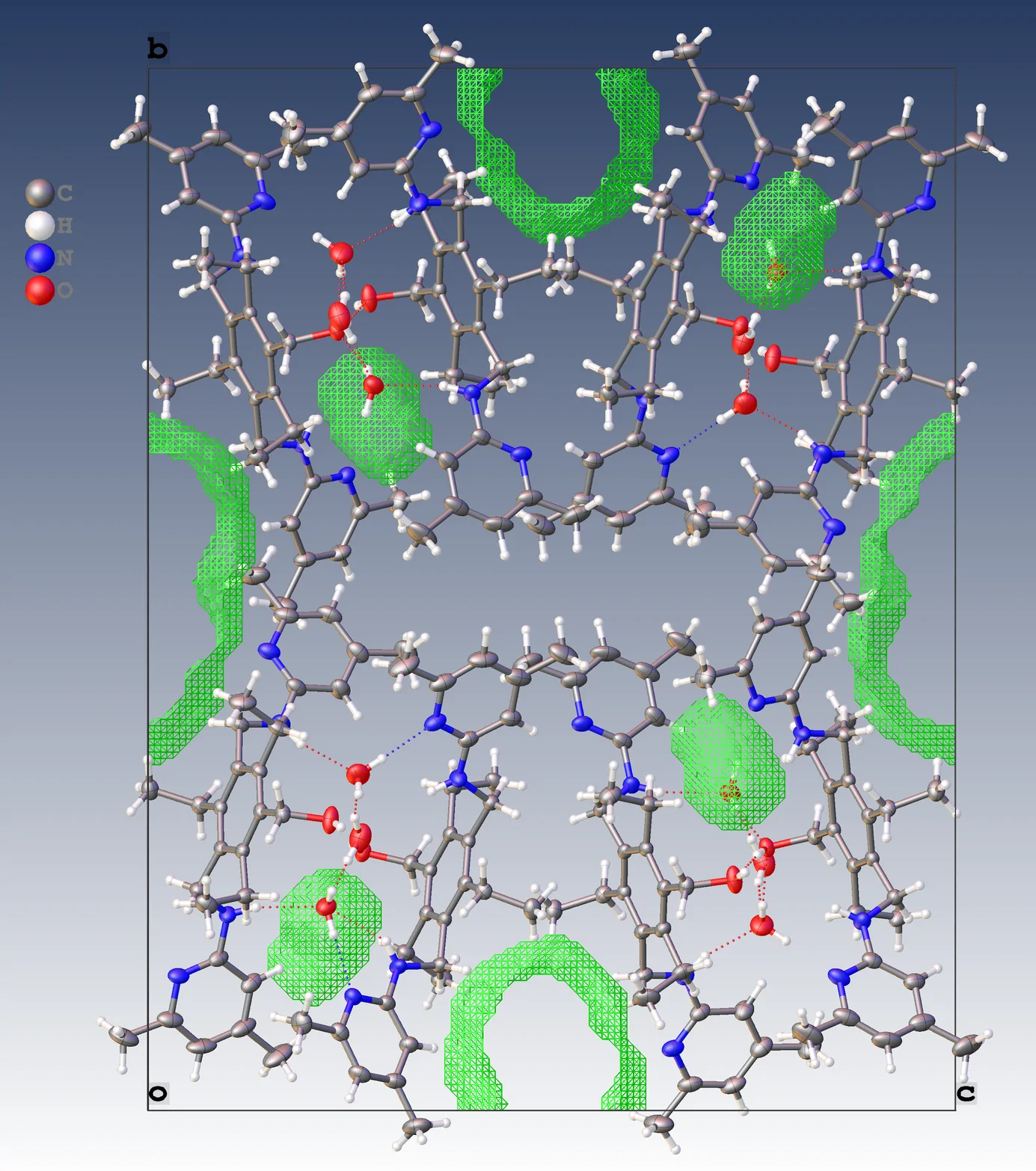
Representation of the surface profile (green mesh) of the crystal voids.

**Table 1:** Hydrogen-bond geometry (Å, °) *Cg*(A) represents the centroid of the C1–C6 ring; *Cg*(B) represents the centroid of the C11–C15/N2 ring; *Cg*(C) represents the centroid of the C21–C25/N4 ring; *Cg*(A′) represents the centroid of the C30–C35 ring; *Cg*(B′) represents the centroid of the C40–C44/N6 ring; *Cg*(C′) represents the centroid of the C50–C54/N8 ring.

*D*—H⋯*A*	*D*—H	H⋯*A*	*D*⋯*A*	*D*—H⋯*A*
N1—H1⋯O5	0.87 (2)	2.03 (2)	2.878 (2)	165 (2)
N3—H3⋯O4	0.86 (1)	2.09 (1)	2.9326 (19)	165 (2)
O1—H1*A*⋯O3^i^	0.84 (1)	1.82 (1)	2.6631 (17)	174 (2)
N5—H5⋯O4^i^	0.87 (1)	2.44 (2)	3.1926 (18)	145 (2)
O2—H2⋯O1	0.84 (1)	1.78 (2)	2.6169 (15)	171 (2)
O3—H3*A*⋯O4	0.86 (1)	1.95 (2)	2.7938 (19)	166 (2)
O3—H3*B*⋯O5	0.85 (1)	1.89 (2)	2.7167 (19)	165 (3)
O4—H4*A*⋯N6^ii^	0.85 (1)	1.94 (1)	2.7718 (17)	167 (2)
O4—H4*B*⋯O2	0.85 (1)	1.93 (2)	2.7426 (15)	161 (2)
O5—H5*A*⋯N8^ii^	0.85 (1)	1.93 (2)	2.7681 (19)	170 (2)
O5—H5*B*⋯O2	0.84 (1)	1.98 (2)	2.7946 (16)	163 (2)
C9—H9*C*⋯*Cg*(B)^i^	0.98	2.84	3.637 (2)	139
C19—H19*A*⋯*Cg*(A’)^iii^	0.98	2.71	3.512 (2)	139
C38—H38*A*⋯*Cg*(B’)^ii^	0.98	2.73	3.612 (2)	150
C45—H45*C*⋯*Cg*(C)^i^	0.98	2.98	3.916 (2)	161
C48—H48*B*⋯*Cg*(A)^iv^	0.98	2.83	3.563 (2)	133
C58—H58*A*⋯*Cg*(C’)^ii^	0.98	2.74	3.613 (2)	149

Symmetry codes: (i) 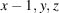; (ii) 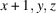; (iii) 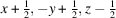; (iv) 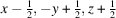.

**Table 2:** Experimental details

Crystal data	
Chemical formula	2C_29_H_40_N_4_O·3H_2_O
*M*_r_	975.34
Crystal system, space group	Monoclinic, *P*2_1_/*n*
Temperature (K)	123
*a*, *b*, *c* (Å)	8.2668 (1), 30.7552 (7), 23.8216 (4)
β (°)	91.142 (1)
*V* (Å^3^)	6055.37 (19)
*Z*	4
Radiation type	Mo *K*α
μ (mm^−1^)	0.07
Crystal size (mm)	0.32 × 0.14 × 0.11 × 0.09 (radius)

Data collection	
Diffractometer	Stoe Stadivari
Absorption correction	Integration (*X-RED32*, Stoe, 2024 ▸)
*T*_min_, *T*_max_	0.982, 0.995
No. of measured, independent and observed [*I* > 2σ(*I*)] reflections	68973, 13195, 10231
*R*_int_	0.028
(sin θ/λ)_max_ (Å^−1^)	0.639

Refinement	
*R*[*F*^2^ > 2σ(*F*^2^)], *wR*(*F*^2^), *S*	0.048, 0.122, 1.02
No. of reflections	13195
No. of parameters	691
No. of restraints	12
H-atom treatment	H atoms treated by a mixture of independent and constrained refinement
Δρ_max_, Δρ_min_ (e Å^−3^)	0.32, −0.23

Computer programs: *X-AREAPilatus3_SV*, *Recipe* and *Integrate* and *X-RED* (Stoe, 2024 ▸), *SHELXT2018/2* (Sheldrick, 2015*a* ▸), *SHELXL2019/3* (Sheldrick, 2015*b* ▸) and *OLEX2* (Dolomanov *et al.*, 2009 ▸).

## supplementary crystallographic information

### (3,5-Bis{[(4,6-dimethylpyridin-2-yl)amino]methyl}-2,4,6-triethylphenyl)methanol sesquihydrate Crystal data

**Table:**

2C_29_H_40_N_4_O·3H_2_O	*F*(000) = 2120
*M**_r_* = 975.34	*D*_x_ = 1.070 Mg m^−^^3^
Monoclinic, *P*2_1_/*n*	Mo *K*α radiation, λ = 0.71073 Å
*a* = 8.2668 (1) Å	Cell parameters from 61413 reflections
*b* = 30.7552 (7) Å	θ = 1.8–29.8°
*c* = 23.8216 (4) Å	µ = 0.07 mm^−^^1^
β = 91.142 (1)°	*T* = 123 K
*V* = 6055.37 (19) Å^3^	Plate, colourless
*Z* = 4	0.32 × 0.14 × 0.11 × 0.09 (radius) mm

### (3,5-Bis{[(4,6-dimethylpyridin-2-yl)amino]methyl}-2,4,6-triethylphenyl)methanol sesquihydrate Data collection

**Table:**

Stoe Stadivari diffractometer	13195 independent reflections
Radiation source: Primux 50 Mo	10231 reflections with *I* > 2σ(*I*)
Graded multilayer mirror monochromator	*R*_int_ = 0.028
Detector resolution: 5.81 pixels mm^-1^	θ_max_ = 27.0°, θ_min_ = 1.8°
rotation method, ω scans	*h* = −10→10
Absorption correction: integration (XRED32, Stoe, 2024)	*k* = −39→39
*T*_min_ = 0.982, *T*_max_ = 0.995	*l* = −30→30
68973 measured reflections	

### (3,5-Bis{[(4,6-dimethylpyridin-2-yl)amino]methyl}-2,4,6-triethylphenyl)methanol sesquihydrate Refinement

**Table:**

Refinement on *F*^2^	Primary atom site location: dual
Least-squares matrix: full	Hydrogen site location: mixed
*R*[*F*^2^ > 2σ(*F*^2^)] = 0.048	H atoms treated by a mixture of independent and constrained refinement
*wR*(*F*^2^) = 0.122	*w* = 1/[σ^2^(*F*_o_^2^) + (0.0499*P*)^2^ + 2.6826*P*] where *P* = (*F*_o_^2^ + 2*F*_c_^2^)/3
*S* = 1.02	(Δ/σ)_max_ = 0.001
13195 reflections	Δρ_max_ = 0.32 e Å^−^^3^
691 parameters	Δρ_min_ = −0.23 e Å^−^^3^
12 restraints	

### (3,5-Bis{[(4,6-dimethylpyridin-2-yl)amino]methyl}-2,4,6-triethylphenyl)methanol sesquihydrate Special details

**Table:**

Geometry. All esds (except the esd in the dihedral angle between two l.s. planes) are estimated using the full covariance matrix. The cell esds are taken into account individually in the estimation of esds in distances, angles and torsion angles; correlations between esds in cell parameters are only used when they are defined by crystal symmetry. An approximate (isotropic) treatment of cell esds is used for estimating esds involving l.s. planes.

### (3,5-Bis{[(4,6-dimethylpyridin-2-yl)amino]methyl}-2,4,6-triethylphenyl)methanol sesquihydrate Fractional atomic coordinates and isotropic or equivalent isotropic displacement parameters (Å^2^)

**Table:**

	*x*	*y*	*z*	*U*_iso_*/*U*_eq_	
C1	0.47235 (16)	0.21319 (5)	0.63944 (6)	0.0234 (3)	
C2	0.55636 (17)	0.17364 (5)	0.63758 (6)	0.0257 (3)	
C3	0.70858 (17)	0.17176 (5)	0.61240 (6)	0.0250 (3)	
C4	0.77677 (16)	0.20923 (4)	0.58941 (6)	0.0220 (3)	
C5	0.69081 (16)	0.24846 (4)	0.59037 (6)	0.0214 (3)	
C6	0.54056 (16)	0.25097 (4)	0.61683 (6)	0.0228 (3)	
C7	0.30872 (17)	0.21518 (5)	0.66683 (6)	0.0273 (3)	
H7A	0.244636	0.239518	0.650716	0.033*	
H7B	0.248771	0.187814	0.659618	0.033*	
C8	0.48126 (19)	0.13303 (5)	0.66188 (7)	0.0353 (4)	
H8A	0.410659	0.141203	0.693264	0.042*	
H8B	0.567864	0.113861	0.677037	0.042*	
C9	0.3816 (2)	0.10826 (6)	0.61740 (9)	0.0465 (5)	
H9A	0.451639	0.099720	0.586589	0.070*	
H9B	0.294535	0.127011	0.602838	0.070*	
H9C	0.334719	0.082216	0.634327	0.070*	
C10	0.80491 (18)	0.12966 (5)	0.61329 (7)	0.0306 (3)	
H10A	0.730775	0.104378	0.612616	0.037*	
H10B	0.874540	0.128029	0.580020	0.037*	
C11	0.98936 (18)	0.09411 (5)	0.68361 (7)	0.0323 (3)	
C12	1.09036 (19)	0.02518 (5)	0.67132 (8)	0.0377 (4)	
C13	1.15631 (19)	0.02462 (5)	0.72469 (8)	0.0405 (4)	
H13	1.213258	−0.000326	0.737845	0.049*	
C14	1.13959 (19)	0.06075 (6)	0.75959 (8)	0.0397 (4)	
C15	1.0574 (2)	0.09595 (6)	0.73805 (7)	0.0378 (4)	
H15	1.046518	0.121562	0.759954	0.045*	
C16	1.1074 (3)	−0.01276 (6)	0.63265 (10)	0.0579 (6)	
H16A	1.000601	−0.021030	0.617563	0.087*	
H16B	1.154856	−0.037329	0.653355	0.087*	
H16C	1.177842	−0.004800	0.601690	0.087*	
C17	1.2031 (2)	0.06128 (7)	0.81926 (9)	0.0554 (5)	
H17A	1.125943	0.046601	0.843544	0.083*	
H17B	1.217435	0.091430	0.831695	0.083*	
H17C	1.307400	0.046140	0.821368	0.083*	
C18	0.94405 (16)	0.20720 (5)	0.56401 (6)	0.0267 (3)	
H18A	1.012420	0.187117	0.586661	0.032*	
H18B	0.993993	0.236424	0.566283	0.032*	
C19	0.94293 (18)	0.19208 (6)	0.50273 (7)	0.0333 (3)	
H19A	0.883362	0.213139	0.479388	0.050*	
H19B	0.890302	0.163591	0.499811	0.050*	
H19C	1.054405	0.189821	0.489791	0.050*	
C20	0.75808 (17)	0.28807 (4)	0.56159 (6)	0.0242 (3)	
H20A	0.816365	0.278931	0.527659	0.029*	
H20B	0.667814	0.307288	0.549522	0.029*	
C21	0.90842 (17)	0.35420 (5)	0.58739 (7)	0.0296 (3)	
C22	0.9024 (2)	0.41272 (5)	0.52749 (9)	0.0416 (4)	
C23	0.9845 (2)	0.43814 (6)	0.56621 (10)	0.0507 (5)	
H23	1.007095	0.467703	0.557851	0.061*	
C24	1.0347 (2)	0.42052 (6)	0.61776 (9)	0.0460 (5)	
C25	0.99841 (19)	0.37787 (5)	0.62811 (8)	0.0378 (4)	
H25	1.033201	0.364406	0.662154	0.045*	
C26	0.8561 (2)	0.42926 (6)	0.47003 (10)	0.0563 (6)	
H26A	0.875287	0.460672	0.468306	0.084*	
H26B	0.741278	0.423309	0.462434	0.084*	
H26C	0.921519	0.414585	0.441875	0.084*	
C27	1.1256 (3)	0.44733 (7)	0.66109 (11)	0.0669 (7)	
H27A	1.212895	0.463292	0.642966	0.100*	
H27B	1.171663	0.428111	0.690067	0.100*	
H27C	1.051292	0.467987	0.678352	0.100*	
C28	0.45255 (18)	0.29408 (5)	0.62098 (7)	0.0296 (3)	
H28A	0.533259	0.317859	0.621873	0.036*	
H28B	0.393772	0.294919	0.656764	0.036*	
C29	0.33193 (19)	0.30222 (6)	0.57234 (8)	0.0382 (4)	
H29A	0.389047	0.301467	0.536700	0.057*	
H29B	0.281338	0.330777	0.576999	0.057*	
H29C	0.248353	0.279622	0.572261	0.057*	
N1	0.90393 (18)	0.12930 (5)	0.66457 (6)	0.0382 (3)	
H1	0.875 (2)	0.1482 (6)	0.6898 (8)	0.057*	
N2	1.00529 (15)	0.05961 (4)	0.65004 (6)	0.0337 (3)	
N3	0.86811 (16)	0.31210 (4)	0.59870 (6)	0.0295 (3)	
H3	0.867 (2)	0.3052 (6)	0.6337 (6)	0.044*	
N4	0.86188 (15)	0.37088 (4)	0.53786 (6)	0.0319 (3)	
O1	0.33128 (13)	0.22139 (5)	0.72605 (5)	0.0410 (3)	
H1A	0.2406 (13)	0.2282 (7)	0.7389 (9)	0.062*	
C30	0.44206 (16)	0.26971 (4)	0.84899 (6)	0.0224 (3)	
C31	0.36855 (16)	0.31078 (4)	0.84497 (6)	0.0234 (3)	
C32	0.21382 (16)	0.31681 (4)	0.86698 (6)	0.0230 (3)	
C33	0.13284 (16)	0.28232 (4)	0.89266 (6)	0.0229 (3)	
C34	0.20841 (16)	0.24165 (4)	0.89664 (6)	0.0223 (3)	
C35	0.36159 (16)	0.23485 (4)	0.87402 (6)	0.0226 (3)	
C36	0.60855 (16)	0.26297 (5)	0.82526 (6)	0.0246 (3)	
H36A	0.672931	0.289805	0.830974	0.030*	
H36B	0.663575	0.239228	0.846208	0.030*	
C37	0.45769 (18)	0.34851 (5)	0.81916 (7)	0.0295 (3)	
H37A	0.378703	0.368347	0.800715	0.035*	
H37B	0.530995	0.337450	0.790072	0.035*	
C38	0.55657 (19)	0.37380 (5)	0.86350 (7)	0.0346 (4)	
H38A	0.609616	0.398582	0.845701	0.052*	
H38B	0.638725	0.354627	0.880408	0.052*	
H38C	0.484514	0.384342	0.892702	0.052*	
C39	0.13181 (17)	0.36062 (5)	0.86177 (6)	0.0262 (3)	
H39A	0.061572	0.365462	0.894358	0.031*	
H39B	0.214330	0.383946	0.861405	0.031*	
C40	−0.05989 (17)	0.39695 (5)	0.79592 (6)	0.0258 (3)	
C41	−0.26181 (17)	0.42474 (5)	0.73751 (7)	0.0309 (3)	
C42	−0.24800 (18)	0.46498 (5)	0.76224 (7)	0.0349 (4)	
H42	−0.315120	0.488249	0.749693	0.042*	
C43	−0.13555 (19)	0.47163 (5)	0.80569 (8)	0.0340 (4)	
C44	−0.04145 (18)	0.43687 (5)	0.82306 (7)	0.0295 (3)	
H44	0.034881	0.440113	0.853116	0.035*	
C45	−0.3812 (2)	0.41600 (6)	0.69036 (8)	0.0432 (4)	
H45A	−0.450415	0.441550	0.684628	0.065*	
H45B	−0.448173	0.390876	0.699886	0.065*	
H45C	−0.322723	0.409890	0.655854	0.065*	
C46	−0.1145 (2)	0.51527 (6)	0.83292 (10)	0.0508 (5)	
H46A	−0.055943	0.511878	0.868836	0.076*	
H46B	−0.220899	0.528139	0.839469	0.076*	
H46C	−0.052805	0.534328	0.808262	0.076*	
C47	−0.03573 (16)	0.28947 (5)	0.91572 (6)	0.0262 (3)	
H47A	−0.095523	0.261585	0.914568	0.031*	
H47B	−0.094797	0.310334	0.891200	0.031*	
C48	−0.03339 (18)	0.30681 (6)	0.97602 (7)	0.0336 (3)	
H48A	0.029238	0.333842	0.977827	0.050*	
H48B	0.016516	0.285183	1.001125	0.050*	
H48C	−0.144431	0.312486	0.987674	0.050*	
C49	0.12224 (17)	0.20453 (5)	0.92486 (6)	0.0251 (3)	
H49A	0.202775	0.184195	0.941466	0.030*	
H49B	0.055188	0.215950	0.955516	0.030*	
C50	−0.05875 (17)	0.14396 (5)	0.89678 (7)	0.0301 (3)	
C51	−0.03800 (19)	0.12342 (5)	0.94899 (7)	0.0342 (4)	
H51	0.028731	0.136114	0.977483	0.041*	
C52	−0.1160 (2)	0.08447 (6)	0.95837 (9)	0.0440 (4)	
C53	−0.2123 (2)	0.06731 (6)	0.91488 (10)	0.0502 (5)	
H53	−0.265754	0.040258	0.919734	0.060*	
C54	−0.2298 (2)	0.08956 (6)	0.86509 (9)	0.0443 (5)	
C55	−0.0965 (3)	0.06219 (7)	1.01440 (10)	0.0623 (6)	
H55A	−0.176859	0.073558	1.040285	0.093*	
H55B	0.012474	0.067692	1.029743	0.093*	
H55C	−0.112362	0.030818	1.009626	0.093*	
C56	−0.3334 (3)	0.07343 (7)	0.81680 (11)	0.0637 (6)	
H56A	−0.267484	0.070929	0.783201	0.096*	
H56B	−0.421998	0.093968	0.809598	0.096*	
H56C	−0.378068	0.044874	0.826134	0.096*	
C57	0.44155 (18)	0.19044 (5)	0.87741 (7)	0.0287 (3)	
H57A	0.506784	0.185902	0.843502	0.034*	
H57B	0.356679	0.167737	0.877816	0.034*	
C58	0.55071 (19)	0.18539 (5)	0.92963 (7)	0.0354 (4)	
H58A	0.601338	0.156577	0.929460	0.053*	
H58B	0.485826	0.188527	0.963379	0.053*	
H58C	0.634879	0.207825	0.929511	0.053*	
N5	0.03485 (16)	0.36201 (4)	0.80992 (6)	0.0301 (3)	
H5	0.004 (2)	0.3376 (5)	0.7948 (8)	0.045*	
N6	−0.16924 (14)	0.39081 (4)	0.75401 (5)	0.0277 (3)	
N7	0.02003 (16)	0.18156 (4)	0.88432 (6)	0.0321 (3)	
H7	−0.001 (2)	0.1937 (6)	0.8516 (7)	0.048*	
N8	−0.15559 (15)	0.12782 (4)	0.85572 (6)	0.0344 (3)	
O2	0.60453 (12)	0.25235 (3)	0.76643 (4)	0.0280 (2)	
H2	0.5120 (12)	0.2450 (7)	0.7544 (9)	0.060 (7)*	
O3	1.03595 (15)	0.23831 (5)	0.76301 (6)	0.0519 (3)	
H3A	0.989 (3)	0.2602 (5)	0.7474 (10)	0.078*	
H3B	0.965 (2)	0.2191 (6)	0.7560 (11)	0.078*	
O4	0.83665 (14)	0.30448 (4)	0.72071 (5)	0.0374 (3)	
H4A	0.819 (2)	0.3308 (3)	0.7297 (9)	0.056*	
H4B	0.7527 (15)	0.2899 (6)	0.7280 (9)	0.056*	
O5	0.79655 (16)	0.17779 (4)	0.76018 (6)	0.0475 (3)	
H5A	0.799 (3)	0.1627 (7)	0.7899 (6)	0.071*	
H5B	0.722 (2)	0.1965 (6)	0.7612 (10)	0.071*	

### (3,5-Bis{[(4,6-dimethylpyridin-2-yl)amino]methyl}-2,4,6-triethylphenyl)methanol sesquihydrate Atomic displacement parameters (Å^2^)

**Table:**

	*U*^11^	*U*^22^	*U*^33^	*U*^12^	*U*^13^	*U*^23^
C1	0.0219 (6)	0.0279 (7)	0.0201 (7)	−0.0037 (5)	−0.0019 (5)	0.0005 (6)
C2	0.0260 (7)	0.0247 (7)	0.0262 (7)	−0.0042 (6)	−0.0016 (6)	0.0047 (6)
C3	0.0252 (7)	0.0231 (7)	0.0266 (7)	0.0000 (6)	−0.0025 (6)	0.0027 (6)
C4	0.0206 (6)	0.0252 (7)	0.0202 (7)	−0.0021 (5)	−0.0030 (5)	0.0008 (5)
C5	0.0223 (6)	0.0219 (7)	0.0199 (7)	−0.0046 (5)	−0.0027 (5)	0.0002 (5)
C6	0.0243 (7)	0.0232 (7)	0.0209 (7)	−0.0012 (5)	−0.0030 (5)	−0.0012 (5)
C7	0.0234 (7)	0.0341 (8)	0.0242 (7)	−0.0035 (6)	−0.0005 (6)	0.0011 (6)
C8	0.0331 (8)	0.0284 (8)	0.0445 (10)	−0.0022 (6)	0.0065 (7)	0.0120 (7)
C9	0.0381 (9)	0.0296 (8)	0.0721 (14)	−0.0112 (7)	0.0057 (9)	0.0022 (9)
C10	0.0310 (8)	0.0247 (7)	0.0361 (9)	0.0019 (6)	0.0002 (6)	0.0054 (6)
C11	0.0268 (7)	0.0307 (8)	0.0395 (9)	0.0070 (6)	0.0070 (6)	0.0092 (7)
C12	0.0275 (7)	0.0253 (8)	0.0605 (12)	0.0000 (6)	0.0028 (8)	0.0075 (7)
C13	0.0288 (8)	0.0316 (8)	0.0612 (12)	0.0058 (7)	−0.0009 (8)	0.0154 (8)
C14	0.0287 (8)	0.0426 (9)	0.0481 (11)	0.0090 (7)	0.0030 (7)	0.0133 (8)
C15	0.0360 (8)	0.0393 (9)	0.0383 (9)	0.0145 (7)	0.0039 (7)	0.0056 (7)
C16	0.0558 (12)	0.0315 (9)	0.0856 (16)	0.0078 (9)	−0.0150 (11)	−0.0047 (10)
C17	0.0512 (11)	0.0611 (13)	0.0535 (12)	0.0236 (10)	−0.0060 (9)	0.0127 (10)
C18	0.0198 (6)	0.0306 (7)	0.0295 (8)	−0.0003 (6)	−0.0020 (6)	0.0028 (6)
C19	0.0275 (7)	0.0434 (9)	0.0291 (8)	0.0041 (7)	0.0033 (6)	0.0025 (7)
C20	0.0255 (7)	0.0217 (7)	0.0254 (7)	−0.0041 (5)	0.0001 (6)	0.0002 (5)
C21	0.0261 (7)	0.0261 (7)	0.0372 (9)	−0.0071 (6)	0.0110 (6)	−0.0065 (6)
C22	0.0334 (8)	0.0266 (8)	0.0654 (12)	−0.0040 (7)	0.0155 (8)	0.0052 (8)
C23	0.0413 (10)	0.0231 (8)	0.0884 (16)	−0.0110 (7)	0.0195 (10)	−0.0050 (9)
C24	0.0388 (9)	0.0366 (9)	0.0632 (13)	−0.0151 (8)	0.0201 (9)	−0.0213 (9)
C25	0.0334 (8)	0.0386 (9)	0.0418 (10)	−0.0135 (7)	0.0129 (7)	−0.0127 (7)
C26	0.0472 (11)	0.0364 (10)	0.0856 (16)	−0.0065 (8)	0.0089 (10)	0.0251 (10)
C27	0.0593 (13)	0.0543 (13)	0.0878 (17)	−0.0304 (10)	0.0193 (12)	−0.0364 (12)
C28	0.0299 (7)	0.0242 (7)	0.0350 (8)	0.0003 (6)	0.0055 (6)	−0.0023 (6)
C29	0.0309 (8)	0.0360 (9)	0.0478 (10)	0.0078 (7)	0.0051 (7)	0.0090 (8)
N1	0.0451 (8)	0.0341 (7)	0.0352 (8)	0.0161 (6)	−0.0022 (6)	0.0032 (6)
N2	0.0259 (6)	0.0279 (7)	0.0474 (8)	0.0018 (5)	0.0046 (6)	0.0069 (6)
N3	0.0327 (7)	0.0273 (6)	0.0283 (7)	−0.0113 (5)	0.0002 (6)	−0.0009 (5)
N4	0.0286 (6)	0.0239 (6)	0.0437 (8)	−0.0052 (5)	0.0093 (6)	0.0020 (6)
O1	0.0248 (5)	0.0730 (9)	0.0253 (6)	−0.0070 (6)	0.0036 (5)	−0.0070 (6)
C30	0.0222 (6)	0.0245 (7)	0.0203 (7)	0.0005 (5)	−0.0017 (5)	−0.0026 (5)
C31	0.0258 (7)	0.0241 (7)	0.0201 (7)	−0.0012 (6)	−0.0025 (5)	−0.0009 (5)
C32	0.0252 (7)	0.0226 (7)	0.0211 (7)	0.0031 (5)	−0.0034 (5)	−0.0014 (5)
C33	0.0208 (6)	0.0256 (7)	0.0220 (7)	0.0006 (5)	−0.0032 (5)	−0.0029 (5)
C34	0.0229 (6)	0.0236 (7)	0.0203 (7)	−0.0016 (5)	−0.0020 (5)	−0.0013 (5)
C35	0.0242 (6)	0.0231 (7)	0.0203 (7)	0.0013 (5)	−0.0026 (5)	−0.0027 (5)
C36	0.0234 (7)	0.0278 (7)	0.0226 (7)	0.0016 (6)	−0.0007 (6)	−0.0022 (6)
C37	0.0316 (7)	0.0259 (7)	0.0312 (8)	0.0003 (6)	0.0054 (6)	0.0036 (6)
C38	0.0338 (8)	0.0270 (8)	0.0431 (10)	−0.0054 (6)	0.0058 (7)	−0.0023 (7)
C39	0.0277 (7)	0.0234 (7)	0.0274 (8)	0.0034 (6)	−0.0033 (6)	−0.0008 (6)
C40	0.0253 (7)	0.0242 (7)	0.0281 (8)	0.0030 (6)	0.0030 (6)	0.0029 (6)
C41	0.0249 (7)	0.0341 (8)	0.0338 (8)	0.0036 (6)	0.0015 (6)	0.0072 (7)
C42	0.0279 (7)	0.0286 (8)	0.0482 (10)	0.0072 (6)	0.0016 (7)	0.0096 (7)
C43	0.0300 (8)	0.0236 (7)	0.0484 (10)	0.0029 (6)	0.0050 (7)	0.0032 (7)
C44	0.0273 (7)	0.0261 (7)	0.0351 (8)	0.0038 (6)	−0.0018 (6)	−0.0004 (6)
C45	0.0358 (9)	0.0503 (10)	0.0432 (10)	0.0066 (8)	−0.0095 (8)	0.0050 (8)
C46	0.0464 (10)	0.0271 (9)	0.0786 (15)	0.0080 (8)	−0.0082 (10)	−0.0086 (9)
C47	0.0196 (6)	0.0297 (7)	0.0291 (8)	0.0026 (6)	−0.0024 (6)	0.0019 (6)
C48	0.0270 (7)	0.0445 (9)	0.0294 (8)	0.0076 (7)	0.0029 (6)	0.0004 (7)
C49	0.0253 (7)	0.0247 (7)	0.0251 (7)	−0.0011 (6)	0.0000 (6)	−0.0004 (6)
C50	0.0248 (7)	0.0255 (7)	0.0404 (9)	−0.0007 (6)	0.0075 (6)	−0.0068 (6)
C51	0.0313 (8)	0.0277 (8)	0.0441 (10)	0.0011 (6)	0.0100 (7)	0.0010 (7)
C52	0.0359 (9)	0.0306 (8)	0.0662 (13)	0.0038 (7)	0.0201 (9)	0.0073 (8)
C53	0.0374 (9)	0.0269 (8)	0.0871 (16)	−0.0081 (7)	0.0186 (10)	−0.0036 (9)
C54	0.0303 (8)	0.0323 (9)	0.0706 (13)	−0.0056 (7)	0.0120 (8)	−0.0161 (9)
C55	0.0562 (12)	0.0464 (11)	0.0853 (17)	0.0032 (10)	0.0259 (12)	0.0269 (11)
C56	0.0486 (11)	0.0504 (12)	0.0921 (18)	−0.0198 (10)	0.0037 (11)	−0.0270 (12)
C57	0.0311 (7)	0.0237 (7)	0.0315 (8)	0.0035 (6)	0.0071 (6)	−0.0008 (6)
C58	0.0342 (8)	0.0362 (9)	0.0361 (9)	0.0136 (7)	0.0067 (7)	0.0090 (7)
N5	0.0377 (7)	0.0222 (6)	0.0299 (7)	0.0076 (5)	−0.0093 (6)	−0.0026 (5)
N6	0.0265 (6)	0.0277 (6)	0.0288 (7)	0.0032 (5)	0.0010 (5)	0.0034 (5)
N7	0.0362 (7)	0.0297 (7)	0.0302 (7)	−0.0102 (6)	−0.0045 (6)	0.0015 (6)
N8	0.0267 (6)	0.0299 (7)	0.0469 (8)	−0.0046 (5)	0.0051 (6)	−0.0120 (6)
O2	0.0237 (5)	0.0361 (6)	0.0242 (5)	−0.0021 (4)	0.0017 (4)	−0.0055 (4)
O3	0.0323 (7)	0.0768 (10)	0.0463 (8)	0.0052 (6)	−0.0018 (6)	−0.0102 (7)
O4	0.0398 (6)	0.0325 (6)	0.0401 (7)	−0.0074 (5)	0.0078 (5)	−0.0037 (5)
O5	0.0470 (7)	0.0441 (8)	0.0517 (8)	0.0182 (6)	0.0086 (6)	−0.0003 (6)

### (3,5-Bis{[(4,6-dimethylpyridin-2-yl)amino]methyl}-2,4,6-triethylphenyl)methanol sesquihydrate Geometric parameters (Å, º)

**Table:**

C1—C2	1.402 (2)	C31—C32	1.404 (2)
C1—C6	1.4037 (19)	C31—C37	1.512 (2)
C1—C7	1.5147 (19)	C32—C33	1.401 (2)
C2—C3	1.406 (2)	C32—C39	1.5125 (19)
C2—C8	1.5150 (19)	C33—C34	1.4004 (19)
C3—C4	1.3993 (19)	C33—C47	1.5241 (19)
C3—C10	1.520 (2)	C34—C35	1.4017 (19)
C4—C5	1.4006 (19)	C34—C49	1.5107 (19)
C4—C18	1.5217 (19)	C35—C57	1.5189 (19)
C5—C6	1.406 (2)	C36—H36A	0.9900
C5—C20	1.5096 (18)	C36—H36B	0.9900
C6—C28	1.5165 (19)	C36—O2	1.4386 (17)
C7—H7A	0.9900	C37—H37A	0.9900
C7—H7B	0.9900	C37—H37B	0.9900
C7—O1	1.4322 (18)	C37—C38	1.534 (2)
C8—H8A	0.9900	C38—H38A	0.9800
C8—H8B	0.9900	C38—H38B	0.9800
C8—C9	1.532 (3)	C38—H38C	0.9800
C9—H9A	0.9800	C39—H39A	0.9900
C9—H9B	0.9800	C39—H39B	0.9900
C9—H9C	0.9800	C39—N5	1.4594 (18)
C10—H10A	0.9900	C40—C44	1.395 (2)
C10—H10B	0.9900	C40—N5	1.3672 (18)
C10—N1	1.457 (2)	C40—N6	1.3467 (19)
C11—C15	1.404 (2)	C41—C42	1.374 (2)
C11—N1	1.3651 (19)	C41—C45	1.504 (2)
C11—N2	1.337 (2)	C41—N6	1.3482 (19)
C12—C13	1.373 (3)	C42—H42	0.9500
C12—C16	1.495 (3)	C42—C43	1.393 (2)
C12—N2	1.363 (2)	C43—C44	1.381 (2)
C13—H13	0.9500	C43—C46	1.499 (2)
C13—C14	1.396 (3)	C44—H44	0.9500
C14—C15	1.372 (2)	C45—H45A	0.9800
C14—C17	1.506 (3)	C45—H45B	0.9800
C15—H15	0.9500	C45—H45C	0.9800
C16—H16A	0.9800	C46—H46A	0.9800
C16—H16B	0.9800	C46—H46B	0.9800
C16—H16C	0.9800	C46—H46C	0.9800
C17—H17A	0.9800	C47—H47A	0.9900
C17—H17B	0.9800	C47—H47B	0.9900
C17—H17C	0.9800	C47—C48	1.532 (2)
C18—H18A	0.9900	C48—H48A	0.9800
C18—H18B	0.9900	C48—H48B	0.9800
C18—C19	1.532 (2)	C48—H48C	0.9800
C19—H19A	0.9800	C49—H49A	0.9900
C19—H19B	0.9800	C49—H49B	0.9900
C19—H19C	0.9800	C49—N7	1.4536 (19)
C20—H20A	0.9900	C50—C51	1.402 (2)
C20—H20B	0.9900	C50—N7	1.3628 (19)
C20—N3	1.4571 (18)	C50—N8	1.346 (2)
C21—C25	1.413 (2)	C51—H51	0.9500
C21—N3	1.3652 (19)	C51—C52	1.381 (2)
C21—N4	1.336 (2)	C52—C53	1.398 (3)
C22—C23	1.378 (3)	C52—C55	1.506 (3)
C22—C26	1.503 (3)	C53—H53	0.9500
C22—N4	1.354 (2)	C53—C54	1.374 (3)
C23—H23	0.9500	C54—C56	1.504 (3)
C23—C24	1.398 (3)	C54—N8	1.348 (2)
C24—C25	1.369 (2)	C55—H55A	0.9800
C24—C27	1.510 (3)	C55—H55B	0.9800
C25—H25	0.9500	C55—H55C	0.9800
C26—H26A	0.9800	C56—H56A	0.9800
C26—H26B	0.9800	C56—H56B	0.9800
C26—H26C	0.9800	C56—H56C	0.9800
C27—H27A	0.9800	C57—H57A	0.9900
C27—H27B	0.9800	C57—H57B	0.9900
C27—H27C	0.9800	C57—C58	1.530 (2)
C28—H28A	0.9900	C58—H58A	0.9800
C28—H28B	0.9900	C58—H58B	0.9800
C28—C29	1.534 (2)	C58—H58C	0.9800
C29—H29A	0.9800	N5—H5	0.869 (14)
C29—H29B	0.9800	N7—H7	0.878 (15)
C29—H29C	0.9800	O2—H2	0.843 (5)
N1—H1	0.870 (15)	O3—H3A	0.859 (5)
N3—H3	0.860 (14)	O3—H3B	0.849 (5)
O1—H1A	0.842 (5)	O4—H4A	0.849 (5)
C30—C31	1.4042 (19)	O4—H4B	0.847 (5)
C30—C35	1.401 (2)	O5—H5A	0.846 (5)
C30—C36	1.5125 (19)	O5—H5B	0.844 (5)
			
C2—C1—C6	120.20 (13)	C35—C30—C36	119.84 (12)
C2—C1—C7	119.74 (13)	C30—C31—C32	119.31 (13)
C6—C1—C7	120.06 (13)	C30—C31—C37	120.29 (13)
C1—C2—C3	119.83 (13)	C32—C31—C37	120.36 (12)
C1—C2—C8	119.75 (13)	C31—C32—C39	119.80 (13)
C3—C2—C8	120.41 (13)	C33—C32—C31	120.64 (12)
C2—C3—C10	120.15 (13)	C33—C32—C39	119.54 (12)
C4—C3—C2	120.27 (13)	C32—C33—C47	119.75 (12)
C4—C3—C10	119.47 (13)	C34—C33—C32	119.29 (12)
C3—C4—C5	119.64 (12)	C34—C33—C47	120.95 (13)
C3—C4—C18	119.88 (12)	C33—C34—C35	120.83 (13)
C5—C4—C18	120.48 (12)	C33—C34—C49	119.44 (12)
C4—C5—C6	120.51 (12)	C35—C34—C49	119.72 (12)
C4—C5—C20	119.74 (12)	C30—C35—C34	119.32 (12)
C6—C5—C20	119.73 (12)	C30—C35—C57	120.05 (12)
C1—C6—C5	119.46 (13)	C34—C35—C57	120.62 (13)
C1—C6—C28	120.12 (13)	C30—C36—H36A	109.0
C5—C6—C28	120.43 (12)	C30—C36—H36B	109.0
C1—C7—H7A	109.8	H36A—C36—H36B	107.8
C1—C7—H7B	109.8	O2—C36—C30	113.05 (11)
H7A—C7—H7B	108.3	O2—C36—H36A	109.0
O1—C7—C1	109.25 (11)	O2—C36—H36B	109.0
O1—C7—H7A	109.8	C31—C37—H37A	109.3
O1—C7—H7B	109.8	C31—C37—H37B	109.3
C2—C8—H8A	109.4	C31—C37—C38	111.50 (13)
C2—C8—H8B	109.4	H37A—C37—H37B	108.0
C2—C8—C9	111.38 (14)	C38—C37—H37A	109.3
H8A—C8—H8B	108.0	C38—C37—H37B	109.3
C9—C8—H8A	109.4	C37—C38—H38A	109.5
C9—C8—H8B	109.4	C37—C38—H38B	109.5
C8—C9—H9A	109.5	C37—C38—H38C	109.5
C8—C9—H9B	109.5	H38A—C38—H38B	109.5
C8—C9—H9C	109.5	H38A—C38—H38C	109.5
H9A—C9—H9B	109.5	H38B—C38—H38C	109.5
H9A—C9—H9C	109.5	C32—C39—H39A	109.8
H9B—C9—H9C	109.5	C32—C39—H39B	109.8
C3—C10—H10A	110.2	H39A—C39—H39B	108.2
C3—C10—H10B	110.2	N5—C39—C32	109.47 (12)
H10A—C10—H10B	108.5	N5—C39—H39A	109.8
N1—C10—C3	107.68 (13)	N5—C39—H39B	109.8
N1—C10—H10A	110.2	N5—C40—C44	121.47 (13)
N1—C10—H10B	110.2	N6—C40—C44	122.20 (13)
N1—C11—C15	118.02 (15)	N6—C40—N5	116.32 (13)
N2—C11—C15	122.83 (14)	C42—C41—C45	121.91 (14)
N2—C11—N1	119.14 (15)	N6—C41—C42	122.02 (14)
C13—C12—C16	121.34 (16)	N6—C41—C45	116.07 (14)
N2—C12—C13	123.11 (16)	C41—C42—H42	120.0
N2—C12—C16	115.55 (17)	C41—C42—C43	119.95 (14)
C12—C13—H13	120.0	C43—C42—H42	120.0
C12—C13—C14	119.93 (15)	C42—C43—C46	121.54 (15)
C14—C13—H13	120.0	C44—C43—C42	118.23 (15)
C13—C14—C17	122.26 (15)	C44—C43—C46	120.23 (16)
C15—C14—C13	117.35 (17)	C40—C44—H44	120.4
C15—C14—C17	120.35 (17)	C43—C44—C40	119.14 (14)
C11—C15—H15	120.0	C43—C44—H44	120.4
C14—C15—C11	119.97 (17)	C41—C45—H45A	109.5
C14—C15—H15	120.0	C41—C45—H45B	109.5
C12—C16—H16A	109.5	C41—C45—H45C	109.5
C12—C16—H16B	109.5	H45A—C45—H45B	109.5
C12—C16—H16C	109.5	H45A—C45—H45C	109.5
H16A—C16—H16B	109.5	H45B—C45—H45C	109.5
H16A—C16—H16C	109.5	C43—C46—H46A	109.5
H16B—C16—H16C	109.5	C43—C46—H46B	109.5
C14—C17—H17A	109.5	C43—C46—H46C	109.5
C14—C17—H17B	109.5	H46A—C46—H46B	109.5
C14—C17—H17C	109.5	H46A—C46—H46C	109.5
H17A—C17—H17B	109.5	H46B—C46—H46C	109.5
H17A—C17—H17C	109.5	C33—C47—H47A	108.9
H17B—C17—H17C	109.5	C33—C47—H47B	108.9
C4—C18—H18A	108.8	C33—C47—C48	113.19 (12)
C4—C18—H18B	108.8	H47A—C47—H47B	107.8
C4—C18—C19	113.77 (12)	C48—C47—H47A	108.9
H18A—C18—H18B	107.7	C48—C47—H47B	108.9
C19—C18—H18A	108.8	C47—C48—H48A	109.5
C19—C18—H18B	108.8	C47—C48—H48B	109.5
C18—C19—H19A	109.5	C47—C48—H48C	109.5
C18—C19—H19B	109.5	H48A—C48—H48B	109.5
C18—C19—H19C	109.5	H48A—C48—H48C	109.5
H19A—C19—H19B	109.5	H48B—C48—H48C	109.5
H19A—C19—H19C	109.5	C34—C49—H49A	109.6
H19B—C19—H19C	109.5	C34—C49—H49B	109.6
C5—C20—H20A	109.3	H49A—C49—H49B	108.1
C5—C20—H20B	109.3	N7—C49—C34	110.16 (12)
H20A—C20—H20B	108.0	N7—C49—H49A	109.6
N3—C20—C5	111.40 (12)	N7—C49—H49B	109.6
N3—C20—H20A	109.3	N7—C50—C51	121.66 (14)
N3—C20—H20B	109.3	N8—C50—C51	122.59 (14)
N3—C21—C25	118.71 (15)	N8—C50—N7	115.75 (15)
N4—C21—C25	123.16 (14)	C50—C51—H51	120.5
N4—C21—N3	118.13 (13)	C52—C51—C50	119.01 (17)
C23—C22—C26	122.07 (16)	C52—C51—H51	120.5
N4—C22—C23	122.53 (18)	C51—C52—C53	117.95 (18)
N4—C22—C26	115.37 (17)	C51—C52—C55	119.73 (19)
C22—C23—H23	119.9	C53—C52—C55	122.31 (17)
C22—C23—C24	120.12 (16)	C52—C53—H53	120.0
C24—C23—H23	119.9	C54—C53—C52	120.01 (16)
C23—C24—C27	121.60 (18)	C54—C53—H53	120.0
C25—C24—C23	117.87 (17)	C53—C54—C56	122.97 (17)
C25—C24—C27	120.5 (2)	N8—C54—C53	122.50 (17)
C21—C25—H25	120.5	N8—C54—C56	114.53 (19)
C24—C25—C21	118.97 (18)	C52—C55—H55A	109.5
C24—C25—H25	120.5	C52—C55—H55B	109.5
C22—C26—H26A	109.5	C52—C55—H55C	109.5
C22—C26—H26B	109.5	H55A—C55—H55B	109.5
C22—C26—H26C	109.5	H55A—C55—H55C	109.5
H26A—C26—H26B	109.5	H55B—C55—H55C	109.5
H26A—C26—H26C	109.5	C54—C56—H56A	109.5
H26B—C26—H26C	109.5	C54—C56—H56B	109.5
C24—C27—H27A	109.5	C54—C56—H56C	109.5
C24—C27—H27B	109.5	H56A—C56—H56B	109.5
C24—C27—H27C	109.5	H56A—C56—H56C	109.5
H27A—C27—H27B	109.5	H56B—C56—H56C	109.5
H27A—C27—H27C	109.5	C35—C57—H57A	109.1
H27B—C27—H27C	109.5	C35—C57—H57B	109.1
C6—C28—H28A	108.9	C35—C57—C58	112.55 (12)
C6—C28—H28B	108.9	H57A—C57—H57B	107.8
C6—C28—C29	113.52 (13)	C58—C57—H57A	109.1
H28A—C28—H28B	107.7	C58—C57—H57B	109.1
C29—C28—H28A	108.9	C57—C58—H58A	109.5
C29—C28—H28B	108.9	C57—C58—H58B	109.5
C28—C29—H29A	109.5	C57—C58—H58C	109.5
C28—C29—H29B	109.5	H58A—C58—H58B	109.5
C28—C29—H29C	109.5	H58A—C58—H58C	109.5
H29A—C29—H29B	109.5	H58B—C58—H58C	109.5
H29A—C29—H29C	109.5	C39—N5—H5	118.6 (13)
H29B—C29—H29C	109.5	C40—N5—C39	122.12 (12)
C10—N1—H1	114.7 (14)	C40—N5—H5	114.5 (13)
C11—N1—C10	124.27 (14)	C40—N6—C41	118.44 (13)
C11—N1—H1	116.4 (15)	C49—N7—H7	119.0 (13)
C11—N2—C12	116.76 (15)	C50—N7—C49	122.79 (13)
C20—N3—H3	116.5 (13)	C50—N7—H7	117.9 (13)
C21—N3—C20	120.87 (13)	C50—N8—C54	117.87 (16)
C21—N3—H3	115.6 (13)	C36—O2—H2	113.3 (15)
C21—N4—C22	117.27 (15)	H3A—O3—H3B	99 (2)
C7—O1—H1A	107.0 (15)	H4A—O4—H4B	108 (2)
C31—C30—C36	119.58 (12)	H5A—O5—H5B	111 (2)
C35—C30—C31	120.58 (12)		
			
C1—C2—C3—C4	0.3 (2)	C30—C31—C32—C33	−0.1 (2)
C1—C2—C3—C10	176.34 (13)	C30—C31—C32—C39	−178.64 (12)
C1—C2—C8—C9	90.87 (18)	C30—C31—C37—C38	−90.02 (16)
C1—C6—C28—C29	−87.45 (17)	C30—C35—C57—C58	86.73 (17)
C2—C1—C6—C5	2.5 (2)	C31—C30—C35—C34	−1.9 (2)
C2—C1—C6—C28	−177.53 (13)	C31—C30—C35—C57	179.19 (13)
C2—C1—C7—O1	84.99 (16)	C31—C30—C36—O2	−85.91 (16)
C2—C3—C4—C5	−1.6 (2)	C31—C32—C33—C34	0.5 (2)
C2—C3—C4—C18	177.93 (13)	C31—C32—C33—C47	−179.37 (13)
C2—C3—C10—N1	−88.80 (16)	C31—C32—C39—N5	90.78 (16)
C3—C2—C8—C9	−88.12 (17)	C32—C31—C37—C38	87.68 (16)
C3—C4—C5—C6	3.3 (2)	C32—C33—C34—C35	−1.7 (2)
C3—C4—C5—C20	−175.19 (12)	C32—C33—C34—C49	179.32 (12)
C3—C4—C18—C19	84.73 (17)	C32—C33—C47—C48	−86.63 (16)
C3—C10—N1—C11	170.47 (14)	C32—C39—N5—C40	176.29 (13)
C4—C3—C10—N1	87.31 (17)	C33—C32—C39—N5	−87.76 (16)
C4—C5—C6—C1	−3.8 (2)	C33—C34—C35—C30	2.3 (2)
C4—C5—C6—C28	176.21 (13)	C33—C34—C35—C57	−178.75 (13)
C4—C5—C20—N3	−86.80 (16)	C33—C34—C49—N7	87.69 (16)
C5—C4—C18—C19	−95.77 (16)	C34—C33—C47—C48	93.46 (16)
C5—C6—C28—C29	92.54 (16)	C34—C35—C57—C58	−92.17 (16)
C5—C20—N3—C21	−162.33 (13)	C34—C49—N7—C50	173.00 (13)
C6—C1—C2—C3	−0.7 (2)	C35—C30—C31—C32	0.8 (2)
C6—C1—C2—C8	−179.73 (13)	C35—C30—C31—C37	178.52 (13)
C6—C1—C7—O1	−94.00 (16)	C35—C30—C36—O2	93.18 (15)
C6—C5—C20—N3	94.65 (15)	C35—C34—C49—N7	−91.33 (15)
C7—C1—C2—C3	−179.73 (13)	C36—C30—C31—C32	179.88 (12)
C7—C1—C2—C8	1.3 (2)	C36—C30—C31—C37	−2.4 (2)
C7—C1—C6—C5	−178.53 (12)	C36—C30—C35—C34	179.02 (12)
C7—C1—C6—C28	1.5 (2)	C36—C30—C35—C57	0.1 (2)
C8—C2—C3—C4	179.26 (14)	C37—C31—C32—C33	−177.83 (13)
C8—C2—C3—C10	−4.7 (2)	C37—C31—C32—C39	3.6 (2)
C10—C3—C4—C5	−177.67 (13)	C39—C32—C33—C34	179.08 (12)
C10—C3—C4—C18	1.8 (2)	C39—C32—C33—C47	−0.84 (19)
C12—C13—C14—C15	−0.6 (2)	C41—C42—C43—C44	−0.6 (2)
C12—C13—C14—C17	177.35 (17)	C41—C42—C43—C46	178.71 (17)
C13—C12—N2—C11	0.8 (2)	C42—C41—N6—C40	−0.2 (2)
C13—C14—C15—C11	2.2 (2)	C42—C43—C44—C40	1.3 (2)
C15—C11—N1—C10	−169.53 (15)	C44—C40—N5—C39	15.1 (2)
C15—C11—N2—C12	0.8 (2)	C44—C40—N6—C41	1.0 (2)
C16—C12—C13—C14	179.39 (17)	C45—C41—C42—C43	−179.87 (16)
C16—C12—N2—C11	−179.49 (15)	C45—C41—N6—C40	179.72 (14)
C17—C14—C15—C11	−175.83 (16)	C46—C43—C44—C40	−178.05 (16)
C18—C4—C5—C6	−176.15 (12)	C47—C33—C34—C35	178.24 (13)
C18—C4—C5—C20	5.32 (19)	C47—C33—C34—C49	−0.8 (2)
C20—C5—C6—C1	174.74 (12)	C49—C34—C35—C30	−178.65 (12)
C20—C5—C6—C28	−5.3 (2)	C49—C34—C35—C57	0.3 (2)
C22—C23—C24—C25	−0.5 (3)	C50—C51—C52—C53	0.1 (2)
C22—C23—C24—C27	179.70 (17)	C50—C51—C52—C55	−179.36 (16)
C23—C22—N4—C21	−1.7 (2)	C51—C50—N7—C49	−4.0 (2)
C23—C24—C25—C21	−1.9 (2)	C51—C50—N8—C54	−2.5 (2)
C25—C21—N3—C20	171.93 (13)	C51—C52—C53—C54	−1.3 (3)
C25—C21—N4—C22	−0.9 (2)	C52—C53—C54—C56	−179.08 (17)
C26—C22—C23—C24	−175.71 (17)	C52—C53—C54—N8	0.7 (3)
C26—C22—N4—C21	176.57 (15)	C53—C54—N8—C50	1.3 (2)
C27—C24—C25—C21	177.87 (16)	C55—C52—C53—C54	178.13 (17)
N1—C11—C15—C14	177.81 (16)	C56—C54—N8—C50	−178.97 (15)
N1—C11—N2—C12	−179.36 (14)	N5—C40—C44—C43	177.27 (15)
N2—C11—C15—C14	−2.4 (2)	N5—C40—N6—C41	−177.88 (13)
N2—C11—N1—C10	10.6 (2)	N6—C40—C44—C43	−1.5 (2)
N2—C12—C13—C14	−0.9 (3)	N6—C40—N5—C39	−166.03 (13)
N3—C21—C25—C24	−177.92 (15)	N6—C41—C42—C43	0.1 (2)
N3—C21—N4—C22	179.76 (14)	N7—C50—C51—C52	−177.41 (14)
N4—C21—C25—C24	2.8 (2)	N7—C50—N8—C54	176.79 (14)
N4—C21—N3—C20	−8.7 (2)	N8—C50—C51—C52	1.9 (2)
N4—C22—C23—C24	2.4 (3)	N8—C50—N7—C49	176.61 (13)

### (3,5-Bis{[(4,6-dimethylpyridin-2-yl)amino]methyl}-2,4,6-triethylphenyl)methanol sesquihydrate Hydrogen-bond geometry (Å, º)

*Cg*(A) represents the centroid of the C1–C6 ring; *Cg*(B) represents the centroid of the C11–C15/N2 ring; *Cg*(C) represents the centroid of the C21–C25/N4 ring; *Cg*(A') represents the centroid of the C30–C35 ring; *Cg*(B') represents the centroid of the C40–C44/N6 ring; *Cg*(C') represents the centroid of the C50–C54/N8 ring.

**Table:**

*D*—H···*A*	*D*—H	H···*A*	*D*···*A*	*D*—H···*A*
N1—H1···O5	0.87 (2)	2.03 (2)	2.878 (2)	165 (2)
N3—H3···O4	0.86 (1)	2.09 (1)	2.9326 (19)	165 (2)
O1—H1*A*···O3^i^	0.84 (1)	1.82 (1)	2.6631 (17)	174 (2)
N5—H5···O4^i^	0.87 (1)	2.44 (2)	3.1926 (18)	145 (2)
O2—H2···O1	0.84 (1)	1.78 (2)	2.6169 (15)	171 (2)
O3—H3*A*···O4	0.86 (1)	1.95 (2)	2.7938 (19)	166 (2)
O3—H3*B*···O5	0.85 (1)	1.89 (2)	2.7167 (19)	165 (3)
O4—H4*A*···N6^ii^	0.85 (1)	1.94 (1)	2.7718 (17)	167 (2)
O4—H4*B*···O2	0.85 (1)	1.93 (2)	2.7426 (15)	161 (2)
O5—H5*A*···N8^ii^	0.85 (1)	1.93 (2)	2.7681 (19)	170 (2)
O5—H5*B*···O2	0.84 (1)	1.98 (2)	2.7946 (16)	163 (2)
C9—H9*C*···*Cg*(B)^i^	0.98	2.84	3.637 (2)	139
C19—H19*A*···*Cg*(A')^iii^	0.98	2.71	3.512 (2)	139
C38—H38*A*···*Cg*(B')^ii^	0.98	2.73	3.612 (2)	150
C45—H45*C*···*Cg*(C)^i^	0.98	2.98	3.916 (2)	161
C48—H48*B*···*Cg*(A)^iv^	0.98	2.83	3.563 (2)	133
C58—H58*A*···*Cg*(C')^ii^	0.98	2.74	3.613 (2)	149

Symmetry codes: (i) *x*−1, *y*, *z*; (ii) *x*+1, *y*, *z*; (iii) *x*+1/2, −*y*+1/2, *z*−1/2; (iv) *x*−1/2, −*y*+1/2, *z*+1/2.
